# Human papillomavirus vaccination: the population impact

**DOI:** 10.12688/f1000research.10691.1

**Published:** 2017-06-12

**Authors:** Lai-yang Lee, Suzanne M. Garland

**Affiliations:** 1 Department of Microbiology and Infectious Diseases, Royal Women’s Hospital, Parkville, Victoria, Australia; 2 Department of Microbiology, Royal Children’s Hospital, Parkville, Victoria, Australia; 3 Department of Infection and Immunity, Murdoch Childrens Research Institute, Royal Children’s Hospital, Victoria, Australia; 4 Department of Obstetrics Gynaecology, University of Melbourne, Parkville, Victoria, Australia

**Keywords:** HPV vaccination, prophylactics, cervical cancer, prevention

## Abstract

We currently have the knowledge and experience to prevent much of human papillomavirus (HPV)-related disease burden globally. In many countries where prophylactic HPV vaccination programs have been adopted as highly effective public health programs with good vaccine coverage, we are already seeing, in real-world settings, reduction of vaccine-related HPV-type infections, genital warts and cervical pre-cancers with potential reductions in vulvar, vaginal and anal pre-cancers. Moreover, we are seeing a change in cervical screening paradigms, as HPV-based screening programs now have strong evidence to support their use as more sensitive ways to detect underlying cervical abnormalities, as compared with conventional cervical cytology. This article describes the impact of prophylactic vaccination on these outcomes and in settings where these vaccines have been implemented in national immunisation programs. Given the successes seen to date and the availability of essential tools, there has been a global push to ensure that every woman has access to effective cervical screening and every girl has the opportunity for primary prevention through vaccination. A gender-neutral approach by offering vaccination to young boys has also been adopted by some countries and is worthy of consideration given that HPV-related cancers also affect males. Furthermore, vaccination of young boys has the advantage of reducing the risk of HPV transmission to sexual partners, lowering the infectious pool of HPV in the general population and ultimately HPV-related diseases for both genders. Therefore, it is appropriate that all countries consider and promote national guidelines and programs to prevent HPV-related diseases.

## Introduction

### Human papillomavirus infection and disease association

Human papillomavirus (HPV) is the commonest sexually transmitted infection, and the resultant diseases have significant morbidity and mortality ^1^. Although most HPV infections are transient, persistent infections are a prerequisite for pre-cancerous lesions and ultimately cancer. ^2– 5^. It is highly likely that latent infection (as infection dormant in the basal cells not readily detectable by diagnostic assays, but reversible) ^6^ or subclinical infections (or both) exist and this is particularly relevant when an individual is immunocompromised since it increases the risk of developing symptomatic disease. Oncogenic HPVs were shown by Harald zur Hausen to be the causative agent of cervical cancer in the early 1980s by virtue of molecular epidemiology; for this work, zur Hausen shared the Nobel Prize in Physiology or Medicine in 2008 ^7^. Of the many genotypes specifically infecting the anogenital area, HPV16 and HPV18 are the commonest high-risk or oncogenic genotypes in cervical cancer and are responsible for approximately 50% of high-grade cervical dysplasias and 70% of cases of cervical cancer, the fourth most common cancer in females globally ^8, 9^. Oncogenic HPVs cause almost 100% of cervical cancers, 90% of anal, 70% of vaginal, 40% of vulvar, 50% of penile and 13% to 72% of oropharyngeal cancers, and HPV16 predominates in all of these non-cervical HPV-related cancers. HPV6 and HPV11, which are classified as low-risk genotypes, cause 90% of genital warts as well as the rare but debilitating recurrent respiratory papillomatosis (RRP) ^10, 11^.

### Prophylactic human papillomavirus vaccines

Given the heavy disease burden of cervical cancer, prophylactic HPV vaccines were developed to target the commonest high- and low-risk HPV genotypes. Two such vaccines were first licensed for clinical use in 2006 following phase 3 clinical trials, which showed efficacy, safety and immunogenicity against vaccine-related HPV types ^12– 15^. Currently available vaccines now include bivalent (2vHPV and targets HPV 16/18) ^12, 13^, quadrivalent (4vHPV and targets HPV 16/18/6/11) ^14, 15^ and nonavalent (targets HPV 16/18/6/11 as well as the five next most common oncogenic types found in cervical cancers, 31/33/45/52/58) vaccines ^16^. Whilst many countries have licensed these vaccines, worldwide introduction into national immunisation programs differs by country, and some vaccines are available only on a user-pays basis and therefore entail an out-of-pocket expense. This has contributed to the resultant generally poor and inequitable uptake. For the greatest impact of these vaccines, the aim is to vaccinate adolescent females prior to sexual debut and to gain high coverage of the target population ^9^. Some countries have implemented catch-up programs for older females or routine vaccination of adolescent males (or both) to increase overall population coverage and enhance herd protection ^17^.

### Vaccine effectiveness and impact

Clinical trials indicated efficacy of the vaccines against the HPV types included in the respective vaccines as well as some modest cross-protection against some non-vaccine but phylogenetically related strains (HPV 31, 33 for HPV16-related types and HPV45 for 18-related types) ^12, 18^. The true benefit of vaccination can be seen only from real-life impact and effectiveness once vaccination has been included in public health programs. To appreciate this requires good surveillance and observation of changes in genoprevalence within vaccine-eligible age cohorts as well as of disease outcomes. Some of the challenges here have included the fact that HPV cannot be cultured by traditional means, so DNA typing in the general community before and after vaccination is required to determine molecular genoprevalence. Although a number of assays to determine the presence of HPV are currently available ^19^, their appropriateness is based on whether they are meant to be used for clinical needs (to detect underlying disease in which case assays are designed with clinical cut-offs and are less sensitive) or for pure epidemiological purposes (these use analytical viral endpoints and are highly sensitive) ^20, 21^. Furthermore, as HPV infection is not notifiable, nor are many of the associated clinical manifestations in most countries, individual surveillance systems have been required to measure the impact and effectiveness of the vaccines. In addition, as the times from infection to the various disease manifestations differ and may be weeks to months (genital warts) to years (pre-cancers) or decades (cancer), the impact of vaccination on each disease varies. Consequently, in the 10 years since the introduction of both 4vHPV and 2vHPV vaccines, measures of vaccine impact have largely been determined by individual observational studies in countries with higher vaccine coverage, although a number of large, countrywide surveillance activities are now occurring. Thus, a direct comparison between the multiple studies published to date is complicated as the population studied, the duration of study and the endpoints observed or measured (or both) differ. Monitoring impact is difficult, particularly as some endpoints such as pre-cancerous lesions require a robust pre-cancer screening program to be in place or are not notifiable or have a prolonged interval between infection and development of diseases, particularly for cancers which usually occur after decades of persistent infection with a high-risk HPV type.

In this article, we define vaccine impact as the effect of public health programs of HPV vaccines at a population level and the measurement thereof of reduction in disease burden (before and after initiation of a vaccination program) and largely focus on this. In contrast, vaccine effectiveness is examined at an individual level ^22^. It is largely observational and determines the effect of vaccination observed in populations after a program commences and compares outcomes in those vaccinated with those unvaccinated.

Vaccine impact will be determined by the type of vaccine program (an ongoing routine vaccination program to a specific age group versus inclusion of a catch-up program which may be limited in time and/or target a narrow or wide number of age cohorts), vaccine coverage, which population is targeted (for example, a program focused entirely on females, a gender-neutral approach, or targeted vaccination such as the HIV-positive population), the duration of vaccine protection, the duration of follow-up after vaccination, and type of surveillance, if any, of various outcome measures.

### Vaccine impact on human papillomavirus infection

Australia was one of the first countries to implement a fully government-funded, population-based HPV vaccination program. It commenced in 2007 as an ongoing school-based program with a three-dose course of the 4vHPV vaccine, targeting females in the first year of high school at age 12 to 13 years, with a catch-up for those ages 12 to 26 years until December 2009 ^23^. In 2013, the Australian government extended the program to include 12- to 13-year-old males, including a 2-year catch-up vaccination program for those ages 14 to 15 years ^24, 25^. Overall, the program has been well received and high vaccine coverage rates have been achieved; the greatest rates have been from those within the school-based cohorts: over 70% have received all three doses for young girls and just under 70% have received all three doses for boys ^24– 26^. Consequently, Australian researchers were among the first to report reductions in the prevalence of vaccine-type HPV infections, by 86% in 18- to 24-year-olds who had received three vaccine doses and 76% for those who had received one or two doses ^22, 27, 28^. In general, Tabrizi *et al*. found that the greatest decline in vaccine-type HPV prevalence corresponded to the age group which had the highest vaccine coverage, even in the unvaccinated but eligible-age population, highly suggestive of herd protection ^28^. Furthermore, Australia is already seeing herd protection in young males as a result of the female program ^29^.

Other countries that have achieved high coverage with 4vHPV or 2vHPV vaccines also report rapid and large declines in vaccine-related HPV infections. For example, in Scotland, which also has a school-based program, which targets 12- to 13-year-old girls as an ongoing program and had a limited 3-year catch-up from 2008 to 2011 for 13- to 17-year-olds, coverage rates for the 2vHPV vaccine have been even greater, at up to 90%, and reported declines in vaccine-related infections were from 29.8% to 13.6% ^30^. In contrast, in the USA, coverage of the 4vHPV vaccine has been much lower, at 40% for females and 22% for males; yet despite this, national impact studies from the USA 4 years after the introduction of HPV vaccines noted a 56% decrease in HPV vaccine types (from cervical-vaginal samples from 14- to 19-year-olds) ^31, 32^. Although the early vaccination initiation uptake rate was low (at 17%) for females 19 to 26 years old in 2009, by 2012 the uptake in the USA had doubled to 34%, resulting in a further 45% decrease in the prevalence of vaccine-type HPV ^33^, or an overall 64% decrease in vaccine-type HPV prevalence after 6 years ^32^. A less marked, but still significant, reduction of 34% prevalence over the same time frame was noted in the 20- to 24-year-old group ^32^. In sexually active US women ages 14 to 24 in the post-vaccination era, those who had received at least one HPV vaccine dose had a 2.1% reduction of vaccine-type HPV prevalence compared with 16.9% in the same age group of unvaccinated women ^32^.

### Genital warts

Genital warts are a common condition, which affects up to 10% of the female population under 45 years old and which usually develops 2 to 3 months after infection with low-risk HPV genotypes, primarily HPV 6 and 11 ^10, 34^. Australia was the first to report a reduction in genital warts, and the reduction was larger and faster than had been expected by researchers ^35, 36^, and the 4vHPV vaccine provided up to 92% reduction in HPV-associated genital warts ^37^. Interestingly, observations from early use of 2vHPV vaccines noted an unexpected decrease in genital warts. It is speculated that this response is due to vaccination resulting in a cell-mediated immune response which confers a moderate amount of protection against some low-risk HPV types ^38, 39^.

Since the commencement of 4vHPV vaccination, there has been a substantial reduction in reports of genital warts in countries with 4vHPV vaccination programs ( Table 1) ^35– 37, 40– 43^. A Belgian study noted a decrease of 8.1% in genital warts in the general population, and the highest decreases were in 16- to 22-year-old males and females between the pre- and post-vaccine era ^44^. Australia, with its high vaccine coverage rates, reported the greatest worldwide decline, of up to 92% in the under-21 age group ^37^. Several studies have highlighted that the greatest efficacy of vaccination against genital warts was when the first dose of vaccine was given at a younger age ^44^ with less of an effect at an older age ^40^.

**Table 1.** Summary of human papillomavirus vaccination programs, outcomes and cervical screening programs.

	Australia	USA	United Kingdom	Denmark	Scotland
Delivery route	School	Clinic	Mostly school	Clinic	School
HPV vaccine program commencement	2007	2006	2008	2009	2008
Vaccine type	4vHPV Ongoing	4vHPV 2006-2016 9vHPV December 2016	2vHPV 2008-2012 4vHPV 2012	4vHPV	2vHPV 2008-2012 4vHPV 2012
Three-dose schedule	Yes				
Two-dose schedule ^a^	No	Yes October 2016	Yes September 2014	Yes 2014	Yes 2014
Females	Yes	Yes	Yes	Yes	Yes
Target age, years	12-13	11-12	12-13	12	12-13
Males (routine)	Yes from 2013	Yes from 2011	No	No	No
Target age males, years	12-13	11-12	-	-	-
Catch-up program	12-26 ♀ 2007-2009 14-15 years old ♂ 2013-2015	13-26 ♀ Ongoing	Up to 18 ♀ 2009-2011	13 -15 ♀ 2009 2nd catch-up 20-27 ♀ 2012	13-17 ♀ 2008-2011
Estimated coverage	2015	2015	2013/2014	2015	
Three doses	77.4% ♀ 66.4% ♂	41.9% ♀ 28.1% ♂	86.7%	82%	12-13 years old 90% Catch-up cohort 66%
At least one dose	85.6% ♀ 77% ♂	62.8% ♀ 49.8% ♂	91.1%	90%	>90%
Vaccine-type HPV infection reduction	18-26 years old 3x dose 86% 2x dose 76%	2010 14-19 ♀ 56% 2012 14-19 years old ♀ 64% 20-24 ♀ 34%	16-18 years old HPV 16/18 prevalence reduce from 19.1% to 6.5%	Not available	HPV 16/18 prevalence reduce from 29.8% to 13.6%
Genital warts (types 6/11) Reduction	Up to 92%	<21 years old 34.8% >21 years old 10%	2vHPV 20.8% 4vHPV- 38.9% ♀ 30.2% ♂	2013 <18 ♀ up to 55.1% <18 years old ♂ up to 36.6%	Since 4vHPV vaccine use, decline in wart prescription (personal communication, Kevin Pollock, 5 January 2017)
CIN/ Adenocarcinoma Reduction	Low-grade 34% High-grade 47% <20 years old 54% 20-24 years old 37%	HPV16/18 CIN2+ ♀vaccinated >24 months before PAP versus unvaccinated ♀, adjusted prevalence ratio of 0.67	Not available	Atypia <18 years old 33.4% (annual percentage) 18-20 years old 12.6% CIN2+ 18-20 years old 14%	CIN1 29% CIN2 50% CIN3 55%
Screening program: Type and interval age commencement	Cervical cytology 18-69 every 2 years	Cervical cytology 21-65 every 3 years	Cervical cytology 25-49 every 3 years 50-64 every 5 years January 2016: UK National Screening Committee recommend HPV primary screening (to commence in the near future) ^90^	Cervical cytology 23-49 every 3 years 50-65 every 5 years	Cervical cytology 25-49 every 3 years 50-64 every 5 years
Comments	1 May 2017 to be changed to HPV DNA every 5 years for 25-74 years old		Since 2014, two-dose schedule <15 years old >15 years old on initiation, three- dose schedule	Since 2014, two-dose schedule <15 years old >15 years old on initiation, three-dose schedule	Screening prior to 2013, 20-60 years old every 3 years
Additional references	^25, 91^	^92– 96^	^97– 100^	^101, 102^	^103, 104^

^a^Less than 15 years at the time of first dose: two-dose regimen of a prime and a boost separated by a minimum of 6 months ^73^.

2vHPV, bivalent human papillomavirus vaccine; 4vHPV, quadrivalent human papillomavirus vaccine; 9vHPV, nonavalent human papillomavirus vaccine; CIN, cervical intraepithelial neoplasia; HPV, human papillomavirus; PAP, Papanicolaou test.

### Cervical cytology and histological abnormalities

Genital warts have a short incubation period and hence this was the first disease reduction noted from vaccination. The second disease outcome to see reductions was cervical dysplasia, which has an intermediate incubation period (peak age of 26 to 30 years) ^45^. Again, this was first reported in an ecological study in Victoria, Australia ^46^ and was made possible because Australia had a HPV vaccine register, comprehensive cervical cytology reporting as well as the ability (following legislation) for the registers to be linked. Australia’s national HPV vaccination programs have led to a 34% decline in low-grade and a 47% decline in high-grade cervical intraepithelial neoplasia (CIN) and adenocarcinoma *in situ*, and the largest reductions were in the vaccinated younger age group ^22, 47^. Women under the age of 20 had a prevalence decline from 10.9/1000 screened women to 5.0/1000 over a period of 10 years, and prevalence in 20- to 24-year-olds decreased from 21.5/1000 screened to 13.5/1000 in a similar time period. In the over-30-year-old age group, the prevalence of high-grade CIN has continued to slowly rise ^48^. Similar declines have been observed in Scotland and Denmark ^49– 52^ ( Table 1). There are randomised controlled phase 2/3 trials indicating that, despite diverse populations and geographical locations, HPV vaccines covering HPV16/18 provided some cross-protection against non-vaccine strain HPV and could protect women against cervical and non-cervical HPV-related infections ^53^.

### Cervical cancer

At present, it is too early to show clinical disease or population-based disease reduction for cervical cancer, as this is a disease peaking in the mid-30s and mid-40s in Australia ^54^, although the peak has shifted to the late 20s to early 30s in some countries ^55^. One would expect to see reductions within the next few years in the under-30-year-olds, particularly in those countries with high coverage of catch-up programs extending to 26 years of age, such as Australia and Denmark.

Based on the PATRICIA trial (Papilloma Trial against Cancer in young Adults) of the 2vHPV vaccine, it has been estimated that with 50% vaccine coverage, there could be a worldwide reduction of cervical cancer incidence of 246,086 cases annually but that with 90% coverage, there could be up to 442,955 cases averted ^56^. With the greater coverage provided by the nonavalent vaccine, it is proposed that with high coverage, 90% to 93% of all cervical cancers would be prevented ^57, 58^.

The incidence of non-cervical HPV-related cancers is generally low and peaks at a later age compared with cervical cancer ^59^. Thus, more time and large population-based effectiveness studies with various cancers as endpoints will be required before impact can be determined. There are early data sets currently reviewing the implication of vaccine-type HPV reduction and the effect on persistent anal infections and intraepithelial neoplasia ^60^.

### Impediments and challenges for the future

***Vaccine uptake and coverage.*** Although the effect of population-wide vaccination has been observed for 10 years, the reported findings may not accurately reflect future impact and this is for several reasons. Some countries have been slow to adopt HPV vaccination or vaccine coverage has been low (or both), even in high-income countries. Vaccine uptake rates in 13- to 17-year-olds in the USA in 2013 were 57% (one dose) and 38% (three doses) ^32^. In Australia, between 2007 and 2015, vaccine uptake rates were 85.6% (one dose) and 77.4% (three doses) for females turning 15 years of age ^61^. Successful strategies to increase coverage have included publically supported health promotion and missed-doses catch-up vaccine campaigns ^62^, client reminders, recall programs, provider assessment and feedback interventions. Low- and middle-income countries often have a high HPV disease burden: however, only 15% of these countries have adopted a vaccination program ^18^. Focusing on these countries and giving reassurance regarding vaccine safety will substantially impact HPV vaccination rates and outcomes ^63^.

***Safety.*** Concern regarding vaccine safety is an issue that often arises. The US Centers for Disease Control and Prevention (CDC), through the Vaccine Adverse Event Reporting System, classified only 7% of adverse events as serious from over 90 million doses of HPV vaccines. The CDC found no causal association between HPV vaccines and ovarian failure, Guillain-Barré syndrome or postural orthostatic tachycardia syndrome. Overall, HPV vaccines have a good safety profile, but ongoing monitoring will be required as 9vHPV vaccines have been introduced ^64– 67^. In Japan, poor or inappropriate information (or both), including reports on complex regional pain syndrome, has resulted in the government withdrawing support for HPV vaccination programs ^68– 70^. Despite comprehensive independent evaluations of safety ^71, 72^, including endorsement of safety by bodies such as the World Health Organization (WHO) ^73^, the International Papillomavirus Society ^66^ and the CDC ^74^, the Japanese government has continued to refuse to re-implement population-wide vaccination ^69^.

***Ideal age for vaccination.*** In many respects, if the HPV vaccines could be administered in childhood, they could use infrastructure already in place for the Expanded Program on Immunization vaccines, as in general these see high uptake even in those countries with low resources. However, the immunity developed by HPV vaccination needs to be robust for several decades to ensure that infection does not occur when young people become sexually active and therefore to prevent diseases in the longer term. Although some studies have shown detectable neutralising antibodies 10 years after vaccination, there are not yet any data about antibody persistence beyond that ^22^. It is currently believed that the antibody responses made by the vaccines are strong and long-lasting and that only small titres are in fact required for protection ^75^.

Over the last 10 years, there have been changes in sexual behaviour, such as lowered median age of sexual debut, increased exposure from the increase in the number of sexual partners, and choice of orientation of sexual partner; however, these trends vary from country to country. The ability of vaccine programs in different countries to be flexible will allow these changes to be accounted for in the future. As shown in Table 1, different delivery programs/routes for the vaccines result in different outcomes in different countries. A single worldwide approach is not appropriate, and programs need to be tailored to each country’s population.

***Dosing.*** New information regarding number of doses of vaccine necessary for long-lasting immunity is becoming available. The current WHO recommendation is that with respect to HPV-vaccine-related-type antibody responses, two doses of HPV vaccine for females 9 to 14 years old is non-inferior to three doses in adult women as long as the two doses are at least 6 months apart ^73^. This decision was based largely on neutralising antibody outcome and more recent findings of reduced HPV-vaccine-type-related disease outcomes, but again good surveillance systems should be able to monitor impact on disease endpoints with longer time intervals from the point of vaccination. Furthermore, this may help to address concerns regarding reduced efficacy ^76^ to ensure that a third dose could be administered if surveillance shows breakthrough disease. For females older than 15 years, those who are immunocompromised or HIV-positive, a three-dose regimen is still recommended ^73, 77^.

***Gender-neutral approaches.*** Although females have been the primary target of HPV vaccine, extending the program towards males may have some benefit. A 2011 Australian study predicted that, based on the female-only vaccination program, there could be up to a 68% decrease in male HPV infections by 2050 and a 14% decrease in head, neck and anogenital cancers. The effect on oropharyngeal cancers is unclear ^78^. This prediction is based on heterosexual relationships offering herd protection. A high-risk HPV group, men who have sex with men, are less likely to benefit from a female-only vaccination program ^79^. A gender-neutral approach may not only reduce HPV-related diseases in males but also reduce the infection and transmission to females, ultimately reducing the pool of infectious virus in a community. Male vaccination would confer an even greater benefit in settings where female vaccination rates are low. Hence, vaccination of males has recently been incorporated into several national programs. Measuring vaccine effectiveness in the context of gender-neutral programs will bring further challenges ^24^.

***Multivalent human papillomavirus vaccines.*** Some countries have implemented or have plans to commence the use of 9vHPV vaccines to offer protection against the additional five next most common oncogenic HPV types after HPV 16/18 in an effort to further improve health outcomes. This move is supported by studies which indicate potential cost savings and health benefits that could result from universal nonavalent vaccination programs ^16, 80, 81^.

### Alternative vaccine policies: “HPV FASTER” potential to impact disease earlier

Consideration for vaccinating women who are already sexually active but may not have been infected with all HPV types covered in the vaccines could well reduce the burden of disease more rapidly. This concept is based on data from phase 3 trials indicating that 2vHPV and 4vHPV vaccines are efficacious, immunogenic and safe in women ages 26 to 45 years old with a 90% vaccine efficacy in protection against cervical pre-cancer in HPV-DNA-negative women (regardless of serostatus) and 50% vaccine efficacy in women who had previously been exposed to HPV ^82– 84^. Moreover, in trials in younger women, it was shown that vaccines were not efficacious when a woman was HPV-positive for a particular type ^85^, although it was likely for women who were HPV-negative but antibody-positive from natural infection that they would gain efficacy against later disease ^86, 87^. The importance of a coordinated screening and vaccination program cannot be overlooked and this has prompted the HPV-FASTER concept. This protocol intends to offer women up to the age of 45 years HPV vaccination and incorporates HPV testing after the age of 30 for screening, with ongoing follow-up and early management of high-risk HPV-positive women. Further studies will be required to investigate the acceptability and feasibility of such an approach, most appropriate cut-off age of offering vaccination, ideal screening commencement, cessation and interval times to refine the protocol ^88^.

***Surveillance.*** A necessary condition for accurately monitoring the impact and effectiveness of vaccine programs is the existence of diligent and ongoing surveillance ^89^. For HPV, this requires surveillance of HPV vaccination coverage and safety, HPV infection genoprevalence, cervical dysplasia incidence, cervical (as well as other anogenital and oropharyngeal cancer, ideally with genotyping of cancers) cancer registries, as well as surveillance for non-cancer outcomes such as warts and RRPs. Ideally, this would include the ability for the various data sources to be linked. Encouragement in participation in screening programs is essential, particularly as new cervical screening programs that incorporate DNA testing as the primary screen usually have longer intervals between screens compared with those with cervical cytology as primary. Also, the vaccinated population may not recognise that, despite vaccination, there is a need for ongoing monitoring. With so many changes, education of the community, clinicians and women in particular is critical.

## Conclusions

Overall, the implementation of HPV vaccine public health programs has resulted in major decreases in vaccine-type HPV infection prevalence as well as associated disease incidence in countries that have introduced them. Administration of vaccines is ideally done prior to sexual debut for both males and females for the greatest impact. Herd and cross-protection contribute further to the impact of the vaccines. More time for observation is required to determine the effect on cancer rates, as carcinomas develop decades after infection acquisition. Although we have the tools to markedly impact HPV-related disease, we also face the challenge of ensuring that all young people, regardless of their geography, social status, or immunocompromised status, are eligible for and can access these vaccines. To observe the vaccine impact and effectiveness of national HPV vaccination programs, support is required from accurate vaccine uptake surveillance, preferably through vaccine registers in addition to genoprevalence and HPV-related disease endpoint surveillance, including cancer registries and pre-cancer screening programs.
